# Measuring the cognitive costs of the COVID-19 pandemic

**DOI:** 10.1186/s13024-021-00492-x

**Published:** 2021-09-23

**Authors:** Gregory S Day

**Affiliations:** Department of Neurology, Mayo Clinic in Florida, Jacksonville, Florida USA

**Keywords:** COVID-19, SARS-CoV-2, Coronavirus, Pandemic, Mild cognitive impairment, Dementia, Cognitive impairment

## Measuring the Cognitive Costs of the COVID-19 Pandemic

Numbers of positive PCR tests, hospital visits, available intensive care beds and ventilators, and lives lost are frequently used to approximate the costs of the Coronavirus Disease 2019 (COVID-19) pandemic. Yet, as weeks turn to months, and months stretch into years, it is becomingly increasingly apparent that the real costs of the pandemic are only just beginning to be counted. This is especially true when considering the neurological consequences of COVID-19.

Severe acute respiratory syndrome coronavirus 2 (SARS-CoV-2) infection leads to prominent systemic inflammation, with direct virus infiltration and proliferation within the central nervous system limited to select cases.[1, 2] Despite this observation, neurologic and psychiatric symptoms and signs are common in patients with COVID-19, including persistent deficits in memory and attention.[3, 4] The high morbidity and burden associated with caring for these patients exemplifies the need to determine the prevalence of cognitive impairment following SARS-CoV-2, and to identify the factors that influence outcomes in recovering patients. In response to this need, Liu and colleagues present cross-sectional data summarizing cognitive outcomes in older patients evaluated 6-months following hospitalization and treatment at one of three COVID-19-designated hospitals in Wuhan, China.[5].

In total, 1,539 participants with COVID-19 were enrolled between February and April 2020. Participants were 60-years or older, without preexisting subjective or diagnosed dementia, or a family history of dementia. Comparison data were obtained from unaffected spousal controls (n = 466). All participants completed a telephone survey, incorporating validated measures of cognition (*Telephone Interview of Cognitive Status-40 [TICS-40], Chinese-language version*), with family members providing collateral history informing post-COVID-19 cognitive changes. Surprisingly, 35.7 % of patients with severe COVID-19—defined as cases with fever or suspected respiratory infection with objective evidence of respiratory compromise—had clinically meaningful cognitive impairment (mild cognitive impairment or dementia). This number was substantially greater than that observed in uninfected spousal controls (4.3 %; *p* < 0.001). No association was observed between non-severe COVID-19 infections and clinically meaningful declines on the TICS-40, although family members were more likely to score non-severe cases (versus spousal controls) within a range associated with clinically meaningful impairment on standardized questionnaires. In subsequent analyses, age, COVID-19 severity, requirement for intensive care unit admission, delirium, and history of stroke, coronary heart disease, and chronic obstructive pulmonary disease were associated with worse TICS-40 score. Greater years of education and use of high flow oxygen therapy were associated with a better TICS-40 score. Similar associations were reported when longitudinal declines were estimated through informant questionnaires.[5] These findings indicate that cognitive impairment is prevalent in older individuals following hospitalization and treatment for COVID-19.

Closer consideration of patient- and disease-associated factors offers additional insights into the potential drivers of cognitive impairment in recovering COVID-19 patients. Disease severity, intensive care unit admission, and chronic obstructive pulmonary disease may identify patients at the greatest risk of clinically significant hypoxemia, and resultant cerebral hypoxia and ischemia. Vascular risk factors may further compound this risk, explaining the increased odds of cognitive impairment in patients with these historical risk factors.[5] Although neuroimaging results were not reported in this study, microstructural damage and disruption of functional brain integrity were noted in studies performing neuroradiological assessments 3-months following SARS-CoV-2 infection.[6] More obvious cerebrovascular disruption may also occur following SARS-CoV-2 infection. Indeed, arterial and venous cerebrovascular thrombotic events are commonly reported in patients hospitalized with COVID-19, including young patients without traditional vascular risk factors.[7] These findings suggest that SARS-CoV-2 infection contributes to a hyperinflammatory and hypercoagulable state that may, in turn, lead to thromboembolic events, cerebrovascular changes, and neuronal loss in susceptible patients.

Neuroinflammation is a well-established contributor to neuropathology in Alzheimer disease and related dementias. Genetic variants altering microglial and astrocyte activation influence the risk of Alzheimer disease in large cohort studies, while abnormal activation of microglia and astrocytes promotes the formation and spread of amyloid-beta and tau pathology in animal and *in vitro* models (for review, see [8]). Prolonged immune activation may also predispose to hippocampal atrophy, tau accumulation and cognitive deficits in older patients recovering from antibody-mediated encephalitis.[9] It is not surprising, therefore, that pathways associated with neuroinflammation and neurodegeneration are upregulated in biofluids and tissues from patients with COVID-19,[1] or that activated microglia feature prominently within the brains of patients who died due to complications of COVID-19.[2] Acute increases in serum neurofilament light chain—a marker of neuroaxonal loss—are also seen in patients hospitalized with COVID-19, with higher levels predicting more severe disease and worse motor outcomes at discharge.[10] Whether neuroinflammation and microglial activation contribute to acute neuronal loss, chronic neurodegenerative changes, or both, remains to be demonstrated in surviving COVID-19 patients.

Longitudinal studies in recovering patients are needed to provide access to data and tissue specimens that will drive discovery and inform the contributors to post-COVID-19 neurological sequelae. These studies should incorporate serial cognitive and clinical assessments, along with structural and molecular (e.g., amyloid-beta and tau) neuroimaging, and biofluid biomarkers of neuroinflammation and neurodegeneration. It will be especially important to obtain these measures early in the disease course, allowing SARS-CoV-2-associated changes in amyloid-beta, tau, and vascular neuropathology to be appreciated through follow-up. In this way, the COVID-19 pandemic presents a unique opportunity to untangle the complex relationship between neurovascular changes, neuroinflammation, and neurodegeneration in an expanding cohort of patients of all ages exposed to SARS-CoV-2. Future studies encompassing broader geographic areas are also needed to ensure the generalizability of results and to validate prevalence estimates of cognitive impairment in other populations affected at other timepoints. This statement acknowledges that much has changed since early 2020, when the cohort reported by Liu et al. was hospitalized.[5] The emergence of new SARS-CoV-2 variants, advances in treatment of severe COVID-19, and the advent and broad dissemination of effective vaccinations may all modify the response to COVID-19, including the risk of post-infectious neurologic sequelae.

A clearer understanding of the drivers of post-COVID-19 cognitive impairment is integral to the development of testable hypotheses and therapeutic approaches to ameliorate neurovascular changes and neuroinflammation, and to slow or stop neurodegeneration in surviving patients. The benefits of this knowledge are likely to extend beyond patients with COVID-19, with the potential to inform approaches to treatment, and improve long-term outcomes in patients with rare(er) autoimmune brain diseases and the millions of individuals worldwide with Alzheimer disease and related dementias. This possibility offers an unexpected upside to an otherwise devastating and costly worldwide pandemic.

## Data Availability

Not applicable.

## Abbreviations

COVID-19: Coronavirus Disease 2019
SARS-CoV-2: Severe acute respiratory syndrome coronavirus 2
TICS-40: Telephone Interview of Cognitive Status-40
